# Long Non-Coding RNAs Link Oxidized Low-Density Lipoprotein With the Inflammatory Response of Macrophages in Atherogenesis

**DOI:** 10.3389/fimmu.2020.00024

**Published:** 2020-01-30

**Authors:** Youyou Yan, Dandan Song, Junduo Wu, Junnan Wang

**Affiliations:** ^1^Department of Cardiology, Second Hospital of Jilin University, Changchun, China; ^2^Key Laboratory of Myocardial Ischemia, Ministry of Education, Harbin Medical University, Harbin, China; ^3^Department of Clinical Laboratory, Second Hospital of Jilin University, Changchun, China

**Keywords:** atherosclerosis, ox-LDL, lncRNAs, cholesterol accumulation, inflammation

## Abstract

Atherosclerosis is characterized as a chronic inflammatory response to cholesterol deposition in arteries. Low-density lipoprotein (LDL), especially the oxidized form (ox-LDL), plays a crucial role in the occurrence and development of atherosclerosis by inducing endothelial cell (EC) dysfunction, attracting monocyte-derived macrophages, and promoting chronic inflammation. However, the mechanisms linking cholesterol accumulation with inflammation in macrophage foam cells are poorly understood. Long non-coding RNAs (lncRNAs) are a group of non-protein-coding RNAs longer than 200 nucleotides and are found to regulate the progress of atherosclerosis. Recently, many lncRNAs interfering with cholesterol deposition or inflammation were identified, which might help elucidate their underlying molecular mechanism or be used as novel therapeutic targets. In this review, we summarize and highlight the role of lncRNAs linking cholesterol (mainly ox-LDL) accumulation with inflammation in macrophages during the process of atherosclerosis.

## Introduction

Atherosclerosis is the major cause of cardiovascular diseases with complications such as endothelial dysfunction, lipid accumulation, and chronic inflammation (1). Uncontrolled cholesterol deposition and chronic inflammation are two key factors in the pathogenesis of atherosclerosis (2). In addition, there is a link between cholesterol and the innate immune system involving macrophages, which plays a central role at different stages of atherogenesis (3). Low-density lipoprotein (LDL), especially its oxidized form (ox-LDL), plays a crucial role in the initiation and development of atherosclerosis by inducing endothelial cell (EC) dysfunction, attracting monocyte-derived macrophages, and promoting chronic inflammation (4). Macrophages engulf ox-LDL to form lipid-laden foam cells that ultimately release pro-inflammatory cytokines and exacerbate local inflammation (5). High-density lipoprotein (HDL) opposes the process of atherosclerosis by promoting the cellular efflux of cholesterol and reducing inflammation (6). HDL has been proposed to inhibit cellular inflammatory signaling resulting in inhibition of monocyte chemoattractant protein-1 (MCP-1) and CD11b expression and monocyte transmigration (7).

Long non-coding RNAs (lncRNAs) are a group of non-protein-coding RNAs >200 nucleotides in length, which participate in diverse biological processes and pathophysiological conditions including cardiovascular disease (8). Recently, several lncRNAs that interfere with the progress of atherosclerosis have been identified. They also participate in the pathology of atherosclerosis such as EC dysfunction, cholesterol accumulation, and inflammation (9). For example, lncRNA-growth arrest-specific 5 (GAS5) expression was significantly increased in the atherosclerotic plaque and exaggerated inflammation in ox-LDL-treated macrophages (10, 11). LncRNA-DYNLRB2-2 was upregulated in ox-LDL-induced THP-1 macrophage-derived foam cells and could promote cholesterol efflux and inhibit inflammation (12). These lncRNAs might help us understand the molecular mechanism underlying the cholesterol accumulation linked with monocyte/macrophage-mediated inflammation during the development of atherosclerosis.

In this review, we summarize and highlight the role of lncRNAs as the link between cholesterol (mainly ox-LDL) accumulation and inflammation during atherosclerosis. The data summarized in the review provide helpful insights regarding further study of lncRNAs associated with atherosclerosis.

## ox-LDL-Mediated Macrophage Inflammation in Atherosclerosis

Endothelial dysfunction leads to the accumulation of lipoproteins and inflammatory cells, including monocytes, in the arterial wall, where monocytes are activated by LDL and differentiated into macrophages (13). In atherosclerotic lesions, macrophages can engulf ox-LDL to form lipid-laden foam cells that ultimately release pro-inflammatory cytokines such as interleukin-6 (IL-6), MCP-1, and tumor necrosis factor alpha (TNF-α), exacerbating local inflammation (14). Macrophages display high heterogeneity, plasticity, and differentiate into two main phenotypes of macrophages, M1 and M2 macrophages, in atherosclerotic plaques (15). M1 macrophages secrete pro-inflammatory cytokines such as IL-1, IL-6, IL-12, IL-15, IL-18, and TNF-α after ox-LDL uptake (16). M2 macrophages secrete anti-inflammatory cytokines such as IL-4, IL-10, and IL-13, and are found predominantly in stable plaques (17, 18).

## Macrophage Receptors Involved in Lipid Accumulation and Inflammation

In atherosclerosis, the pattern recognition receptors (PRRs) on monocyte-derived macrophages, such as scavenger receptors (SRs), are involved in lipid recognition and inflammation (19). Scavenger receptor-A1 (also known as macrophage SR-A1, MSR1, or CD204) belongs to the class A SR family and facilitates uptake of ox-LDL in macrophages (20). SR-A1 knock-down reduces the formation of foam cells and atherosclerosis progression in ApoE^−/−^ mice (21). CD36 is a member of the SR class B family that can facilitate the endocytosis of ox-LDL in macrophages (22). CD36 and SR-A are principal contributors to cholesterol uptake, accounting for up to 90% of ox-LDL loading in macrophages (23). Lectin-like oxidized low-density lipoprotein receptor-1 (LOX-1) is not detectable in human monocytes, but is induced in differentiated macrophages by several stimuli, including ox-LDL, high glucose levels, and pro-inflammatory cytokines (24). LOX-1 in macrophage uptake and degradation of ox-LDL under normal conditions present in small amounts (25). The ATP-binding cassette transporters A1 and G1 (ABCA1 and ABCG1) are responsible for most of the macrophage cholesterol efflux to the serum or HDL in macrophage foam cells (26). The ABCA1 is a primary cholesterol transporter, and is associated with severe HDL deficiency and premature atherosclerosis (27). ABCA1-deficient mice have low HDL cholesterol and develop foam cell lesions (28). Inhibition of ABCA1 in mouse macrophages led to cholesterol accumulation and increased secretion of pro-inflammatory cytokines (29). Similarly, ABCG1 knock-down resulted in a moderate rise of atherosclerotic plaques in LDL receptor-deficient mice (30). In contrast, inhibition of ABCG1 exerted an atheroprotective effect in LDL receptor-deficient mice (31).

In addition to cholesterol accumulation, these receptors alone or in conjunction with Toll-like receptors (TLRs) participate in macrophage-mediated inflammation (32). For example, TLR2 and TLR4 act as the PRRs for ox-LDL and can directly trigger pro-inflammatory signaling via the upregulation of fatty acid-binding protein aP2 (33). Depletion of aP2 in macrophages reduced production of TNF-a, MCP-1, and IL-6 upon exposure to ox-LDL (34). SR-A1 was also identified as a pro-inflammatory receptor in macrophages (35). SR-A^−/−^ mice exhibit increased sensitivity to endotoxin-induced shock, related to high TNF-α production (36). CD36 has been shown to cooperate with TLR2, TLR4, and TLR6 to induce inflammation in macrophages (37). Ox-LDL triggers MyD88-dependent inflammation through the CD36-TLR4-TLR6 heterodimer in macrophages (38), accompanied by activation of mitogen-activated kinases (MAPKs) such as p38 MAPK or NF-κB (39). LOX-1 is significantly reduced in atherosclerosis and involved in inflammation; NF-κB expression and inflammatory markers are reduced in LOX-1 deficient animals (40). HDL has potent anti-inflammatory effects via regulation of ABCA1 and ABCG1 (41). ABCA1 and ABCG1 deficiency increased inflammatory cytokine production via activation of TLR4 signaling in macrophages (42). In addition, ABCA1 and ABCG1 suppress macrophage inflammatory responses through activation of TLR2 and TLR3 (9, 43, 44). Activation of TLR3 and TLR4 signaling suppresses expression of ABCA1 and ABCG1 in macrophages (43, 44).

There is a loop between cholesterol accumulation and inflammation. Pro-inflammatory cytokines such as TNF-a and IL-6 promote SR-A1 expression by activating NF-κB, which is closely related to the inflammatory immune response (45). Ox-LDL upregulates the expression of CD36 to further enhance its uptake by macrophages via activation of peroxisome proliferator–activated receptor-γ (PPAR-γ) (46). In addition, TLR agonists strongly stimulate particle uptake by macrophage via CD36. Exogenous TLR2 activation enhances CD36-mediated particle uptake. Similarly, ox-LDL or inflammatory cytokines such as TNF-a, IL-1 can upregulate LOX-1 expression (25, 47).

## lncRNA Biology and Function in Atherosclerosis

Long non-coding RNAs (lncRNAs) are RNAs longer than 200 nucleotides in length, with little functional protein-coding ability as they lack open reading frames (ORFs) (48). Although lncRNAs are expressed at lower levels and are evolutionarily ill conserved, they show more tissue-specificity (49). Based on their genomic location, lncRNAs are classified into long intergenic non-coding RNAs, intronic lncRNAs, bidirectional lncRNAs, sense lncRNAs, antisense lncRNAs, and enhancer RNAs (49). According to the molecular mechanism, lncRNAs are classified into four main categories: signals, decoys, guides, and scaffolds (50). Signal lncRNAs can react to diverse stimulations and transduce relevant signals. Decoy lncRNAs can sequester other effectors, such as RNA-binding proteins and microRNAs, to negatively regulate their function. Guide lncRNAs act as molecular chaperones, localizing ribonucleoproteins to specific chromatin targets. Scaffold lncRNAs bind distinct proteins to coordinate their function. In addition, lncRNAs exhibit a unique ability to interact with target molecules and guide them to specific genomic regions in neighboring (cis) or distantly located (trans) genes to modulate gene expression. Finally, lncRNAs can bind partners, acting as a scaffold (50). In addition, lncRNAs can be further categorized into cellular or nuclear lncRNA. Cellular lncRNAs can influence the stability of mRNAs by regulating mRNA translation or interacting partners of microRNAs (51, 52). Nuclear lncRNAs are involved in epigenetic processing and can regulate gene expression at the transcriptional and post-transcriptional levels (51, 52).

**Figure 1:**
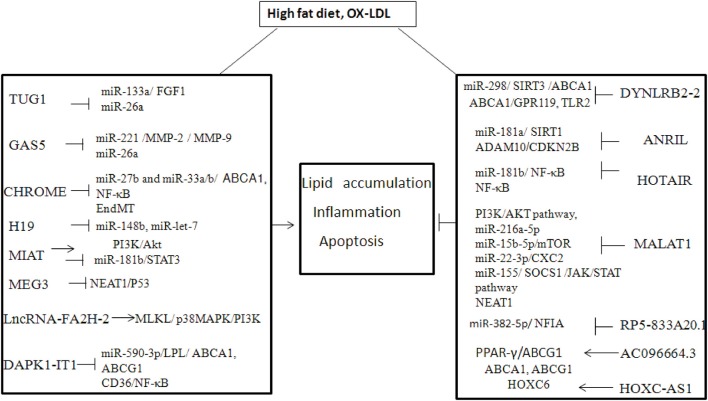
The mechanism of lncRNA in high fat diet or Ox-LDL induced atheroslerosis.

A growing number of studies have found that lncRNAs can influence the progression of atherosclerosis by regulating the function of ECs, vascular smooth muscle cells (VSMCs), vascular inflammation, macrophages, and lipid metabolism (53). The onset of atherosclerosis is partly mediated by EC dysfunction, when subjected to various noxious stimuli (54) For example, lncRNA GAS5 is highly expressed in ECs, and downregulation of lncRNA GAS5 exacerbates hypertension-induced microvascular dysfunction (55). MALAT1 is strongly upregulated in response to hypoxia and induces a switch of ECs from a migratory phenotype to a proliferative phenotype) (56) The proliferation and migration of smooth muscle cells (SMCs) play an important role in atherosclerotic lesion progression and re-stenosis, which are also regulated by lncRNAs (57). It has been shown that lncRNA p21 can repress cell proliferation and stimulate apoptosis in VSMCs by modulating transcriptional activity of p53 both *in vitro* and *in vivo* (58). Another study showed that lncRNA Ang362, induced by angiotensin II, also modulates the proliferation of VSMCs (59). In addition, hypoxia-related lncRNA HIF1a-AS1 markedly inhibited proliferation and promoted apoptosis by decreasing B-cell lymphoma 2 (Bcl2) expression and increasing the expression of caspase3 and caspase8 (or caspase9 in ECs) in VSMCs and ECs (60). Macrophages with high cholesterol accumulation can induce chronic inflammation, which facilitates the development of foam cells and atherosclerotic plaques. Many lncRNAs have been identified in atherosclerotic plaques or ox-LDL-induced macrophage-derived foam cells (9). Several lncRNAs are involved in the regulation of cholesterol metabolism and inflammation (61, 62), which might unveil the molecular mechanism of cholesterol-mediated inflammation (Table 1, Figure 1).

**Table 1:** lncRNA linked the lipid and inflammation in atherosclerosis.

**lncRNA**	**Expression**	**Disease**	**mRNA**	**Function**	**Ref**
DYNLRB2-2	↑	ox-LDL treated THP-1 or Raw264.7 LPS treatment	miR-298/ SIRT3 /ABCA1 ABCA1/GPR119 TLR2	Lipid accumulation (–) Lipid accumulation (–) Inflammation (–) Lipid accumulation (–) Inflammation (–)	(12) (63) (64)
ANRIL	↓	High-fat diet ApoE^−/−^ mice ox-LDL-exposed THP-1 High-fat diet ApoE^−/−^ mice LPS-treated HCAECs HUVECs TNF-α-treated ECs	miR-181a/ SIRT1 ADAM10 CDKN2B miR-181b/ NF-κB NF-κB	Apoptosis of VSMCs (–) Lipid accumulation (–) Inflammation (–) Lipid accumulation (+) Inflammation (+) Inflammation (+)	(65) (66) (67, 68) (69) (70)
TUG1	↑	High-fat diet ApoE^−/−^ mice ox-LDL-exposed THP-1 ox-LDL treated HCAECs	miR-133a/ FGF1 miR-26a	Lipid accumulation (+) Inflammation (+) Apoptosis (+)	(71) (72)
GAS5	↑	ox-LDL treated ECs ox-LDL-treated HAECs, THP-1 cells	miR-221 /MMP-2 / MMP-9, miR-26a	Inflammation (+) Inflammation (+) Apoptosis (+)	(73) (74)
CHROME	↑	CAD patients	miR-27b and miR-33a/b/ ABCA1, NF-κB	Inflammation (+) Inflammation (+)	(75) (76)
MALAT1	↑	CAD patients ox-LDL-treated HUVECs	EndMT PI3K/AKT pathway, miR-216a-5p, miR-15b-5p/mTOR miR-22-3p/CXC 2 miR-155/ SOCS1 /JAK/STAT pathway	Apoptosis (+) Inflammation (+) Inflammation (–) Inflammation (–)	(77–80) (81) (82) (83)
MEG3	↑	ox-LDL-treated HAECs, THP-1 High fat diet ApoE^−/−^ mice ox-LDL-treated HCAECs ox-LDL-treated HCAECs	NEAT1 P53 miR-361-5p/ ABCA1 miR-223 /NLRP3 miR-204/CDKN2A	EC apoptosis (+) Inflammation (+) Pyroptosis (+) Inflammation (+)	(84) (84) (85) (86)
HOTAIR	↑	ox-LDL treated Raw264.7 cells ox-LDL-treated ECs ox-LDL treated RAW264.7	- FXR1 /NF-κB PI3K/Akt	Apoptosis (–) Inflammation (–) Inflammation (–) Inflammation (–)	(82) (83) (84)
MIAT	↑	ox-LDL-treated THP-1 High-fat diet ApoE^−/−^ mice	miR-181b/STAT3	Apoptosis (+)	(87)
H19	↑	ox-LDL-treated HCAMCs ox-LDL-treated HUVECs	miR-148b miR-let-7	Apoptosis (+) Apoptosis (+)	(88) (89)
RP5-833A20.1	↑	ox-LDL-treated HCAMCs	miR-382-5p/ NFIA	Lipid accumulation (–) Inflammation (–)	(90)
HOXC-AS1	↓	ox-LDL-treated	ABCA1, ABCG1 HOXC6	Lipid accumulation (–)	(91)
AC096664.3	↓	ox-LDL-treated Human macrophages	PPAR-γ/ABCG1	Lipid accumulation (–)	(92)
LncRNA-FA2H-2	↓	ox-LDL-treated THP-1 ox-LDL treated THP-1	MLKL/ p38MAPK/PI3K	Autophagy (+) Inflammation (+)	(93)
DAPK1-IT1	↑	High-fat fed ApoE^−/−^ mice ox-LDL treated ECs or SMCs ApoE^−/−^ mice ox-LDL treated THP-1	miR-590-3p/LPL/ ABCA1, ABCG1 CD36/NF-κB	Lipid accumulation (+) Inflammation (+)	(94)

*^↑^, increase; ^↓^, decrease; +, promote; –, inhibit*.

### lncRNA-DYNLRB2-2

LncRNA DYNLRB2-2 was upregulated in ox-LDL induced THP-1 macrophage-derived foam cells and ox-LDL treated THP-1 or RAW264.7 cells *in vitro* (12, 95, 95). DYNLRB2-2 displayed an anti-atherosclerotic property by promoting cholesterol efflux and inhibiting inflammation. DYNLRB2-2 inhibited THP-1 macrophage foam cell formation and promoted cholesterol efflux by increasing ABCA1 expression *in vitro*. DYNLRB2-2 induced ABCA1 expression by sponging miR-298 targeting SIRT3, thereby activating the LKB1/AMPK/mTOR signaling pathway *in vitro* (12). Another study revealed that the lncRNA-DYNLRB2-2 promoted cholesterol efflux and inhibited inflammation in THP-1 macrophage-derived foam cells by regulating ABCA1 and G protein-coupled receptor 119 (GPR119), respectively (63). Moreover, DYNLRB2-2 was also found to promote cholesterol efflux and inhibit the lipopolysaccharide (LPS)-induced inflammatory cytokines including TNF-α, IL-1β, and IL-6 in macrophages by decreasing TLR2 expression (64). Over-expression of TLR2 reversed the effect of DYNLRB2-2 on cholesterol accumulation and inflammation in macrophages or ApoE^−/−^ mice with a high-fat diet (64). These evidences show that DYNLRB2-2 has therapeutic potential in atherosclerosis.

### Anril (CDKN2B-AS1)

LncRNA Antisense non-coding RNA in the INK4 locus (ANRIL), also called CDKN2B antisense RNA 1 (CDKN2B-AS1), has gene polymorphism that is associated with a risk of developing coronary artery disease (CAD) (71, 96–98). For example, the variant of rs10757274 and rs1333049 were associated with the susceptibility of the Han CAD population, indicating that ANRIL might affect the development of atherosclerosis (97). Further study showed that ANRIL could enhance the viability of VSMCs via regulating miR-181a/silent information regulator 1 (SIRT1) (65). In addition, ANRIL downregulation correlated with elevated pro-inflammatory cytokines in patients with acute ischemic stroke, indicating ANRIL modulates inflammation (99). It was suggested that ANRIL could promote cholesterol efflux and inhibit inflammatory cytokines such as IL-1β and TNF-α in ox-LDL-exposed THP-1 macrophages by silencing ADAM (a disintegrin and metalloprotease) 10 (66). ADAMs participated in a variety of metabolic and inflammatory conditions including atherosclerosis, neuro-inflammatory response, and acute lung inflammation (66). In colorectal cancer, ANRIL could sponge miR-Let-7a to enhance the expression of ATP binding cassette subfamily C member 1 (ABCC1), which promotes cholesterol efflux and inhibits inflammation *in vivo* (100). The circular form of ANRIL (circ-ANRIL) was also identified in human atherosclerotic plaques and conferred atheroprotection in vascular smooth muscle cells and macrophages (101). In contrast, ANRIL was also found to promote the lipid uptake and cholesterol transport in THP-1 macrophage-derived foam cells and mouse models by regulating the CDKN2B promoter (67, 68). ANRIL promotes LPS induced-inflammation in human coronary artery endothelial cells (HCAECs) and human umbilical vein endothelial cells (HUVECs) via sponging miR-181b and then activating NF-κB signaling *in vitro* (69). Similarly, ANRIL induced by TNF-α also promoted inflammatory cytokines such as IL-6 or IL-8 in ECs through activation of NF-κB (70). Therefore, the effect of ANRIL on lipid metabolism and inflammation needs further study.

### TUG1

LncRNA taurine-upregulated gene 1 (TUG1) was upregulated in ox-LDL treated ECs, VSMCs, and macrophages and in high-fat fed ApoE^−/−^ mice (102). The high levels of TUG1 correlated with low levels of miR-26a in high-fat diet ApoE^−/−^ mice and a further study confirmed that TUG1 could accelerate the ox-LDL-induced the apoptosis of ECs by sponging miR-26a (72). MiR-26a was also found to attenuate hyperlipidemia and the inflammatory response *in vivo*, suggesting the TUG1 might play an important role in cholesterol efflux and inflammation by regulating of miR-26a (103). It was suggested that TUG1 promoted hyperlipidemia and inflammatory cytokines such as IL-6 and TNF-α by sponging miR-133a, which targeted the fibroblast growth factor 1 (FGF1) in ox-LDL-treated RAW264.7 cells (102).

### lncRNA GAS5

LncRNA GAS5 was significantly increased in the atherosclerotic plaque collected from patients and in the rat model (10, 11, 104). GAS5 was found to accelerate ox-LDL-induced apoptosis of HCAECs and THP-1 cells by sponging miR-26a *in vitro* (74). As discussed above, miR-26a could attenuate hyperlipidemia and the inflammatory response in high fat-fed ApoE^−/−^ mice, indicating that GAS5 might affect the development of atherosclerosis via regulation of cholesterol efflux and inflammation (104). Another study showed that GAS5 could promote monocyte migration and production of inflammatory cytokines including IL-6, IL-1β, TNF-α, and MCP-1 via sponging of miR-221, leading to upregulation of MMP-2 and MMP-9 in ox-LDL treated THP-1 cells (73). Consistently, miR-221 was involved in atherosclerotic development by regulating the function of ECs and inflammation (105, 106). It was also found that GAS5 was downregulated in M2 macrophages (107). These evidences suggest that GAS5 might promote the development of atherosclerosis via regulation of EC function, cholesterol efflux, inflammation, and might be used as a therapeutic target.

### lncRNA CHROME (AC009948.5, linc-OSBPL6)

LncRNA CHROME (cholesterol homeostasis regulator of miRNA expression), also known as AC009948.5 or linc-OSBPL6, was upregulated in the plasma of patients with atherosclerotic vascular disease (75, 108). CHROME has seven variants that are transcriptionally regulated by the cholesterol-sensing liver X receptors, which are also associated with cellular cholesterol homeostasis. CHROME sponges miR-27b and miR-33a/b, which target ABCA1, promoting cholesterol efflux and HDL metabolism by gene analysis (75). CHROME was higher in the plasma of patients with inflammatory conditions such as coronary artery disease and inhibition of CHROME contributed to the decrease of inflammatory gene expression including NF-κB by transcriptome analyses (76). Therefore, CHROME might promote atherosclerosis development by regulating cholesterol efflux and inflammation.

### MALAT1

Long non-coding RNA metastasis-associated lung adenocarcinoma transcript 1 (MALAT1) was first found in non-small cell lung cancer and also found to be upregulated in high-fat diet ApoE^−/−^ mice and patients with unstable angina (109, 110). MALAT1 could protect HUVECs against ox-LDL-induced apoptosis by upregulating endothelial-to-mesenchymal transition (EndMT), which is associated with plaque instability *in vitro* (77). The protective effect of MALAT1 was also found in ox-LDL-treated HCAECs, partly through competing with miR-22-3p, which targeted the CXC chemokine receptor 2 (81). Other studies revealed that the protective effect of MALAT1 in HUVECs was associated with the induction of autophagy by inhibiting the PI3K/AKT pathway or sponging miR-216a-5p, respectively (78, 79). However, another study revealed that MALAT1 repressed the ox-LDL-induced apoptosis by inhibiting autophagy through sponging miR-15b-5p to activate the mTOR signaling pathway *in vitro* (80). In addition, MALAT1 also repressed the production of ox-LDL mediated pro-inflammatory cytokines such as IL-6 and IL-8 in HCAECs via sponging miR-155, which targeted Suppressor of cytokine signaling 1 (SOCS1) and restrained JAK/STAT signaling pathway (82). Exosomal MALAT1 released by ox-LDL-treated HUVECs promoted M2 macrophage polarization and decreased the expression of the M1 macrophage marker, IL-12 (111). In MALAT1-deficient ApoE^−/−^ mice, the pro-inflammatory cytokines such as IFN-γ, IL6, and aortic root plaque size were increased. MALAT1 exerted anti-inflammatory effects by interacting with nuclear enriched abundant transcript (NEAT1), which enhanced lipid uptake in macrophages through the formation of par speckles as well as the inflammatory molecules including IL-6, IL-1β, and TNF-α in ox-LDL-treated RAW264.7 cells (83, 112). Collectively, MALAT1 could inhibit ox-LDL-induced EC apoptosis, inflammation and might be targeted for atherosclerosis treatment.

### MEG3

Long non-coding RNA maternally expressed gene 3 (MEG3) was significantly decreased in ox-LDL-treated VSMCs and the serum of high fat-fed ApoE^−/−^ mice 103). Inhibition of MEG3 protected VSMCs from ox-LDL-induced injury by increasing p53 expression *in vitro* (84). Another study showed that MEG3 promoted the ox-LDL-induced apoptosis in VSMCs by sponging the miR-361-5p, which targeted ABCA1 expression (113). As ABCA1 can regulate cholesterol metabolism and inflammation, indicating MEG3 could indirectly affect cholesterol metabolism and inflammation. It was shown that MEG3 enhanced pyroptosis, a highly inflammatory form of programmed cell death, in ox-LDL treated HCAECs by sponging miR-223 targeting NOD-like receptor protein 3 (NLRP3) (85, 114). Consistently, MEG3 was upregulated in ox-LDL treated RAW264.7 cells and increased secretion of TNF-α and IL1β by sponging miR-204, which targeted the cyclin-dependent kinase inhibitor 2A (CDKN2A) (86). Thus, MEG3 promotes the development of atherosclerosis by enhancing EC dysfunction and inflammation.

### HOTAIR

Long non-coding RNA Hox transcript antisense intergenic RNA (HOTAIR) was much lower in ECs and peripheral blood lymphocytes from atherosclerotic plaques in CAD patients (115). HOTAIR protected ECs against ox-LDL-induced apoptosis (115, 116). In addition, HOTAIR was also significantly increased in ox-LDL-treated RAW264.7 cells and could reduce lipid accumulation and inhibit the inflammatory response by suppressing FXR1 via the NF-κB pathway (117). In contrast, HOTAIR also found to increase the inflammatory cytokines such as IL-6, IL-1β, in ox-LDL treated THP-1 cells by sponging miR-330-5p (118). Consistently, miR-330-5p inhibited inflammation and promoted macrophage M2 polarization in diabetic mice (119). Thus, the effect of HOTAIR on lipid accumulation and inflammation needs further study.

### MIAT

Long non-coding RNA myocardial infarction associated transcript (MIAT), was highly expressed in human carotid plaques, in the serum of patients with vulnerable plaques and high-fat fed ApoE^−/−^ mice (120, 121). MIAT significantly increased lipid content and promoted apoptosis of aortic cells as well as the production of inflammatory cytokines such as IL-1β, IL-6, and TNF-α in ApoE^−/−^ mice through the activation of the PI3K/Akt signaling pathway (121). MIAT promotes the phenotypic transition of SMCs to macrophage-like cells via kruppel-like factor 4 (KLF4) *in vitro* (122). In addition, MIAT inhibited efferocytosis of macrophages by sponging miR-149-5p, which targeted the anti-phagocytic molecule CD47 *in vitro* (123). In contrast, MIAT was also found to inhibit ox-LDL-induced apoptosis in HCAMCs partly by inhibition of miR-181b, which induces the upregulation of signal transducer and activator of transcription 3 (STAT3) *in vitro* (87). Notably, STAT3 is a critical inflammatory mediator in atherosclerosis and inhibition of STAT3 suppressed the ox-LDL induced inflammation in high-fat fed ApoE^−/−^ mice (124). MIAT promotes inflammation and increased lipid content to accelerate atherosclerosis development, however its effect on apoptosis of SMCs needs further study.

### H19

Long non-coding RNA H19 was significantly upregulated in the serum of patients with atherosclerosis and those with a high risk of coronary artery disease in a Chinese population (125, 126). In addition, H19 was also induced by ox-LDL and inhibition of H19 protected HUVECs and HAVSMCs against ox-LDL induced apoptosis via sponging miR-148b or miR-let-7, respectively (88, 89). H19 knockdown suppressed the pro-inflammation cytokines such as IL-1β, IL-6, and TNF-α in HUVECS by sponging miR-let-7 *in vitro* (89). Additionally, H19 was found to regulate lipid metabolism in the liver, suggesting H19 could regulate cholesterol metabolism (127). Knockdown of H19 decreased lipid accumulation and pro-inflammatory factors (TNF-α, IL-1β) and increased the level of anti-inflammatory factors (IL-4, IL-10) in ox-LDL treated RAW264.7 cells by upregulating miR-130b (90). Thus, H19 could link lipid accumulation and inflammation, and knockdown of H19 might be a therapeutic strategy in atherosclerosis.

### RP5-833A20.1

Long non-coding RNA RP5-833A20.1 was upregulated in human acute monocytic leukemia macrophage–derived foam cells or induced by ox-LDL (128). RP5-833A20.1 regulated the cholesterol homeostasis and inhibited inflammatory cytokines (TNF-α, IL-1β, and IL-6) in human acute monocytic leukemia macrophages by reducing expression of nuclear factor I A (NFIA), whose genomic location overlaps with RP5-883A20.1, by upregulating miR-382-5p (128). NFIA could stimulate the expression of ABCA1 and ABCG1 but reduce the expression of SRA1 and CD36 in THP-1 macrophages (128). NFIA could be used to treat atherosclerosis by promoting cholesterol homeostasis and inhibiting inflammatory cytokines.

### lncRNA HOXC-AS1

Long non-coding RNA-HOXC cluster antisense RNA 1 (HOXC-AS1) was significantly downregulated in atherosclerotic plaques and ox-LDL treated THP-1 macrophages (91). HOXC-AS1 can suppress ox-LDL-induced cholesterol accumulation in THP-1 macrophages by promoting homeobox C6 (HOXC6) expression (91).

### lncRNA AC096664.3

Long non-coding RNA AC096664.3 expression was decreased in ox-LDL treated VSMCs and THP-1 macrophages. Downregulation of AC096664.3 caused an increase in total and free cholesterol and decreased ABCG1 by inhibiting the expression of PPAR-γ (92).

### lncRNA FA2H-2

Long non-coding RNA FA2H-2 was significantly decreased in high-fat fed ApoE^−/−^ mice and reduced inflammatory cytokines such as MCP-1 and IL-6 in atherosclerotic lesions. FA2H-2 was also reduced in ox-LDL treated ECs or SMCs and could inhibit the activity of NF-κB, which plays an important role -n inflammation. In addition, FA2H-2 also repressed autophagy by inhibiting mixed lineage kinase domain-like protein (MLKL), which can activate the inflammasome via the p38 MAPK/PI3K pathway (93). FA2H-2 could regulate ox-LDL induced inflammation, involved in the development of atherosclerosis.

### lncRNA DAPK1-IT1

Long non-coding RNA-DAPK1-IT1 was upregulated in high-fat fed ApoE^−/−^ mice and ox-LDL treated macrophages (94). DAPK1-IT1 decreased the levels of HDL and increased the levels of LDL in the mouse model. DAPK1-IT1 reduced ABCA1 and ABCG1 protein levels in THP-1 macrophages by sponging miR-590–3p, which targeted lipoprotein lipase (LPL). Moreover, the DAPK1-IT1/miR590-3p/LPL axis also increased the production of inflammatory cytokine via activation of CD36, and NF-κB (94). Thus, DAPK1-IT1 plays an important role in lipid metabolism and inflammation.

### lncRNA LOC286367 and lncRNA ENST00000602558.1

Long non-coding RNA ENST00000602558.1 was found to promote lipid accumulation in VSMCs, along with downregulation of ABCG1-mediated cholesterol efflux to HDL, through a p65-dependent pathway (129). LncRNA LOC286367 could inhibit ABCA1 expression in macrophages and reduced expression of the LPS-induced pro-inflammatory cytokines including IL-6 and TNF-α. As ABCA1 and ABCG1 play an important role in lipid accumulation and inflammation, these data indicate that lncRNA LOC286367 and lncRNA ENST00000602558.1 link lipid accumulation with inflammation in atherosclerosis (130).

## Discussion

Atherosclerosis is usually recognized as a chronic inflammatory disease arising from unbalanced lipid metabolism. Clinically, statins remain the mainstay of treatment of atherosclerosis by lowering LDL cholesterol. However, statins also exert anti-inflammatory effects, suggesting a link between cholesterol accumulation and chronic inflammation. Scavenger receptors (SRs), the ATP-binding cassette transporters A1 and G1 in macrophages, are involved in cholesterol metabolism alone or in cooperation with TLRs and play an important role in inflammation. However, cholesterol accumulation and chronic inflammation were independently investigated to unveil the pathology of atherosclerosis. Recently, lncRNAs have emerged as critical regulators of atherosclerotic processes including EC dysfunction, lipid accumulation, and inflammation and might be used to unveil the molecular mechanism of cholesterol (mainly ox-LDL) accumulation during inflammation or as targets for therapy (53, 54). For example, DYNLRB2-2 promoted cholesterol efflux and inhibited inflammation via activation of ABCA1 and inhibition of TLR2 (64). However, TUG1 promoted hyperlipidemia and inflammatory cytokines such as IL-6 and TNF-α via sponging miR-133a (103). In addition to unveiling the molecular link between cholesterol accumulation in atherosclerosis, lncRNAs might be used as therapeutic targets. For example, MIAT significantly increased lipid content and promoted apoptosis of aortic cells as well as increased inflammatory cytokines, indicating it might be used as a therapeutic target for atherosclerosis (120–122). Specific antisense oligonucleotides treatment targeting MALAT1 could inhibit the growth of mammary carcinoma or metastasis of human non-small cell lung cancer, respectively (131, 132). Thus, specific antisense oligonucleotide treatment targeting lncRNAs represent possible therapeutic strategies for cholesterol accumulation and inflammation, and might have beneficial effects in patients with atherosclerosis.

## Author Contributions

YY wrote the article. DS and JWu designed the table and figure. JWa provided ideas and the initial design.

### Conflict of Interest

The authors declare that the research was conducted in the absence of any commercial or financial relationships that could be construed as a potential conflict of interest.

## Abbreviations

LDL: Low-density lipoprotein
ox-LDL: oxidized LDL
lncRNAs: Long non-coding RNAs
EC: endothelial cell
HDL: High-density lipoprotein
MCP-1: monocyte chemoattractant protein-1
lncRNA- GAS5: growth arrest-specific 5
IL-6: interleukin-6
TNF-α: tumor necrosis factor alpha
PRRs: pattern recognition receptors
SRs: scavenger receptors
LOX-1: Lectin-like oxidized low-density lipoprotein receptor-1
ABCA1: ATP-binding cassette transporters A1
ABCG1: ATP-binding cassette transporters G1
TLRs: Toll-like receptors
PPAR-γ: peroxisome proliferator–activated receptor-γ
LPS: lipopolysaccharide
SIRT1: silent information regulator 1
ANRIL: Antisense non-coding RNA in the INK4 locus
ADAM: a disintegrin and metalloprotease
HUVECs: human umbilical vein endothelial cells
TUG1: taurine-up-regulated gene 1
CHROME: cholesterol homeostasis regulator of miRNA expression
MALAT1: metastasis-associated lung adenocarcinoma transcript 1
EndMT: endothelial-to-mesenchymal transition
NEAT1: neighboring nuclear enriched abundant transcript
MEG3: maternally expressed gene 3
CDKN2A: cyclin-dependent kinase inhibitor 2A
HOTAIR: Hox transcript antisense intergenic RNA
NLRP3: NOD-like receptor protein 3
MIAT: myocardial infarction associated transcript
STAT3: signal transducer and activator of transcription 3
KLF4: kruppel-like factor 4
SMC: smooth muscle cells
STAT3: signal transducer and activator of transcription 3
LPL: lipoprotein lipase
MLKL: mixed lineage kinase domain-like protein
HOXC6: homeobox C6
HOXC-AS1: HOXC cluster antisense RNA.
